# Complete Genome Sequence of *Sphingopyxis terrae* Strain 203-1 (NBRC 111660), a Polyethylene Glycol Degrader

**DOI:** 10.1128/genomeA.00530-16

**Published:** 2016-06-09

**Authors:** Yoshiyuki Ohtsubo, Shouta Nonoyama, Yuji Nagata, Mitsuru Numata, Keiko Tsuchikane, Akira Hosoyama, Atsushi Yamazoe, Masataka Tsuda, Nobuyuki Fujita, Fusako Kawai

**Affiliations:** a Department of Environmental Life Sciences, Graduate School of Life Sciences, Tohoku University, Sendai, Japan; b Biological Resource Center, National Institute of Technology and Evaluation, Tokyo, Japan; c Center for Fiber and Textile Science, Kyoto Institute of Technology, Kyoto, Japan

## Abstract

The complete genome sequence of *Sphingopyxis terrae* strain 203-1, which is capable of growing on polyethylene glycol, was determined. The genome consisted of a chromosome with a size of 3.98 Mb and a plasmid with a size of 4,328 bp. The strain was deposited to the National Institute of Technology and Evaluation (Tokyo, Japan) under the number NBRC 111660.

## GENOME ANNOUNCEMENT

*Sphingomonas terrae* strain E-1-A, which is capable of growing on polyethylene glycol (PEG) in a mixed culture with an associate strain E-1-B, was isolated from activated sludge and identified as a *Flavobacterium* sp. (1). Later, the strain was identified as the type strain of *Sphingomonas terrae* (2) and deposited to the Institute for Fermentation (Osaka, Japan) (IFO) under the number IFO 15098, which was reidentified as *Sphingopyxis macrogoltabida* (3), based on the taxonomical standards proposed by Yabuuchi et al. (4). Upon the IFO’s closure, the strain was transferred to the National Institute of Technology and Evaluation (Tokyo, Japan) (NITE) and has been stocked under number NBRC 15098. Unfortunately, strain NBRC 15098 lost its polyethylene glycol (PEG)-degrading ability. The genome of strain NBRC 15098 has been sequenced to confirm the loss of PEG-degradative genes, and the results will be reported elsewhere. Fortunately, we could reisolate a PEG-degrading strain from the laboratory stock of F. Kawai, which was identified as *Sphingopyxis terrae* and, for a clear distinction, designated strain 203-1. Surprisingly, the monoculture of strain 203-1 could grow on PEG, which would be beneficial to maintain its degradation ability and to apply the strain to bioremediation and other uses.

For Illumina sequencing, the MiSeq system was used to sequence mate-pair (MP) and PCR-free paired-end libraries for 301 bp each from both ends. Each pair of reads was processed and extracted by ShortReadManager (SRM) to extract MP reads, as well as single-end (SE) reads, and paired-end (designated here as PE1) reads. These reads, together with reads obtained from the paired-end library (designated here as PE2), were loaded to SRM to count the occurrence frequency of 31-mers, and then each read was trimmed to delete parts occurring only once or twice in the entire reads; reads shorter than 150 bp were discarded. We used Newbler version 2.8 to assemble 2 million PE2 reads (corresponds to 433 Mb) and 0.47 million MP reads (96 Mb) to obtain two scaffolds, one with 35 gaps and the other with no gap (corresponds to a circular plasmid).

The finishing was conducted using GenoFinisher and AceFileViewer (5). All 35 gaps were closed by GenoFinisher and AceFileViewer, and the finished sequence was confirmed by FinishChecker. The complete sequence of the genome of strain 203-1 comprised one circular chromosome of 3,979,087 bp and a plasmid with a size of 4,328 bp. The sequences were annotated by the NCBI Prokaryotic Genome Annotation Pipeline (PGAP) and curated using GenomeMatcher (6). While referring to annotation data obtained from the Microbial Genome Annotation Pipeline (http://www.migap.org) (7), we corrected start codon positions and added genes that were missing in the PGAP annotation. The genes involved in PEG degradation (8, 9) were located on the chromosome.

### Nucleotide sequence accession numbers.

The genome sequence of *Sphingopyxis terrae* strain 203-1 has been deposited in NCBI/GenBank under accession numbers CP013342 and CP013343. *Sphingopyxis terrae* strain 203-1 is available from the Biological Resource Center, National Institute of Technology and Evaluation (Tokyo, Japan). Its deposit number is NBRC 111660.
